# Recruitment of Hippocampal and Thalamic Pathways to the Central Amygdala in the Control of Feeding Behavior Under Novelty

**DOI:** 10.21203/rs.3.rs-3328572/v1

**Published:** 2023-09-11

**Authors:** Eliza M. Greiner, Gorica Petrovich

**Affiliations:** Boston College; Boston College

**Keywords:** context, consumption, sex differences, retrograde, pathways, tracing

## Abstract

It is adaptive to restrict eating under uncertainty, such as during habituation to novel foods and unfamiliar environments. However, sustained restrictive eating is a core symptom of eating disorders and has serious long-term health consequences. Current therapeutic efforts are limited, because the neural substrates of restrictive eating are poorly understood. Using a model of feeding avoidance under novelty, our recent study identified forebrain activation patterns and found evidence that the central nucleus of the amygdala (CEA) is a core integrating node. The current study analyzed the activity of CEA inputs in male and female rats to determine if specific pathways are recruited during feeding under novelty. Recruitment of direct inputs from the paraventricular nucleus of the thalamus (PVT), the infralimbic cortex (ILA), the agranular insular cortex (AI), the hippocampal ventral field CA1, and the bed nucleus of the stria terminals (BST) was assessed with combined retrograde tract tracing and Fos induction analysis. The study found that during consumption of a novel food in a novel environment, larger number of neurons within the PVTp and the CA1 that send monosynaptic inputs to the CEA were recruited compared to controls that consumed familiar food in a familiar environment. The ILA, AI, and BST inputs to the CEA were similarly recruited across conditions. There were no sex differences in activation of any of the pathways analyzed. These results suggest that the PVTp-CEA and CA1-CEA pathways underlie feeding inhibition during novelty and could be potential sites of malfunction in excessive food avoidance.

## Introduction

Initial exposure to novel stimuli in the environment induces avoidant behaviors. Animals and people are cautious when trying new foods and when eating in a new environment, and typically restrict their consumption until the new food and environment are deemed to be safe. These behaviors are adaptive, but can persist and lead to psychopathology. Restrictive eating is a core symptom in Anorexia Nervosa and Avoidant/Restrictive Food Intake Disorder. Both disorders co-occur with anxiety and are more common in women than in men (Treasure et al. 2020; Zimmerman and Fisher 2017).

Our previous study compared how novel food and novel feeding environment impact food consumption in male and female rats and found robust effects of context novelty and sex differences. Novel food hypophagia was enhanced in a novel environment in both sexes. During repeated exposures, males and females showed similar habituation to a novel food in a familiar environment. In a novel environment, males habituated to eating a novel food faster than females, who showed suppressed consumption throughout testing (Greiner & Petrovich 2020).

Recently, we begun to identify the neural substrates that control feeding behavior under novelty. We determined activity, with Fos induction, within several key forebrain regions during the consumption of novel or familiar foods in novel or familiar environments and compared patterns in male and female rats. We found that novelty robustly recruited the central nucleus of the amygdala (CEA). Novel food, increased Fos induction in all subregions of the CEA, and novel context increased Fos induction within the capsular region of the CEA. These results indicated that the CEA is involved in processing novelty of both foods and environments. We also found that novel context induced Fos in three regions known to project to the CEA (Greiner et al., 2023; Greiner 2023), the paraventricular thalamus (PVT) (Li & Kirouac, 2008), infralimbic cortex (ILA) (Hurley et al., 1991), and dorsal agranular insular cortex (AId) (McDonald et al., 1996). Additionally, Fos induction within the CEA subregions were differently correlated with Fos induction within other forebrain regions, depending on food and context familiarity. The complex patterns strongly implicated CEA processing and its communication with other forebrain areas in mediating feeding inhibition during novelty processing. The goal of the current study was to establish if such communications are via direct inputs to the CEA.

Here, we examined five major pathways to the CEA. Our analyses focused on the three CEA-input regions identified based on their Fos induction patterns during feeding under novelty (Greiner et al., 2023; Greiner 2023), and their role in the control of feeding behavior: the PVT, ILA and AId. We also examined the inputs from the bed nucleus of the stria terminalis (BST), and the ventral CA1 region of the hippocampal formation. The BST is important in stress, fear and anxiety and it has been implicated in mediating responding to unpredictable stimuli (Walker et al., 2009; Gungor & Pare, 2016; Kim et al., 2013) which is relevant to novelty processing, due to the uncertainty inherent in a novel stimulus. The BST oval (BSTov) and juxtacapsular (BSTju) nuclei send dense projections to subregions of the CEA (Dong et al. 2000; Dong et al., 2001), and activation of the projections from that region (dorsolateral) of the BST to the CEA was shown to increase anxiety-like responding in an elevated plus maze (Yamauchi et al., 2018; Pomrenze et al., 2019). The hippocampus encodes space and contextual information (Wilson & McNaughton, 1993), and detects novel environments (Gomez-Ocadiz et al, 2022), and the CEA receives direct inputs from the ventral field CA1 (Cenquizca & Swanson, 2007). Neurons within CA1 have shown increased firing to the novelty of an object (Larkin et al., 2014) and Projections from the CA1 to the CEA are necessary for the context gated retrieval of cued fear memories (Xu et al., 2016), suggesting that this connection is important for mediating appropriate responding in a context-dependent manner.

Even though these five regions are strong candidates for mediating the effects of novelty on food consumption, their connections with the CEA have not been examined. Additionally, it is completely unknown if these CEA inputs control food consumption under novelty differently in males and females. To address this, the current study used a combination of retrograde tract tracing and Fos induction, to determine whether the PVT, ILA, AI, BST, and CA1 neurons that send direct projections to the CEA are specifically recruited during the consumption test under novelty and whether that activation was sex specific. Male and female rats received injections of the retrograde tracer, FluoroGold (FG), to retrogradely label neurons that send direct projections to the CEA. Rats were then tested for consumption in either familiar or novel condition and Fos induction was assessed within retrogradely-labeled neurons, in order to establish activity in these pathways.

## Materials & Methods

### Subjects

Adult male (n = 24) and female (n = 22) Long Evans rats (Charles River Laboratories; Portage, MI), that weighed 200–250g upon arrival, were individually housed and maintained on a 12-hour light/dark cycle (lights on 06:00). Males and females were housed in the same colony room on separate shelves. After arrival, subjects were allowed one week to acclimate to the colony housing room before surgical procedures began, during which they had *ad libitum* access to water and standard rat chow (Purina Lab Diet Prolab RMH 3000; 3.47 kcal/g; 26% protein, 15% fat, 59% carbohydrates), and were handled daily. All housing and testing procedures were in compliance with the National Institutes of Health Guidelines for Care and Use of Laboratory Animals and approved by the Boston College Institutional Animal Care and Use Committee.

### Surgical Procedure

Animals were deeply anesthetized with isoflurane (5%; Baxter Healthcare Corporation, Deerfield, IL, USA), and while under anesthesia received unilateral (side counterbalanced) stereotaxically placed infusions into the CEA of 0.1μl 4% Fluorogold (FG Fluorochrome LLC, Denver, CO) delivered at a rate of 0.1μl/min (relative to bregma anterior-posterior [AP]:−2.0mm, mediolateral [ML]: +/− 3.8mm, dorsoventral [DV]: −7.5mm). The injector remained in the site for 6 minutes post-infusion to allow for the diffusion of FG. A 10 μl Hamilton syringe with 32 gauge cannula driven by a motorized stereotaxic injector (Stoelting, Wood Dale, IL) was used to deliver microinjections. Stereotaxic surgeries were performed according to the procedures for aseptic technique in survival surgery and postoperative care approved by Boston College IACUC. Behavioral experiments started two weeks after surgery to allow for recovery and sufficient transport of the tracer.

### Apparatus

Half of the animals were tested in a familiar environment (their housing cages) and the other half were tested in a novel environment (behavioral chamber; plexiglass and aluminum box (30×28×s30cm) with grid flooring and a recessed port (3.2×4.2cm) on one wall; Coulbourn Instruments). Each chamber was enclosed in monolithic rigid foam box. Food was presented in a ceramic bowl.

### Behavioral Testing Procedure

After recovery from surgery, male and female rats were tested for consumption of either a novel food in a novel environment or a familiar food in a familiar environment. There were four groups, in order to test the effects of sex and novelty on consumption: female novel, female familiar, male novel, male familiar. All groups underwent one 30-minute testing session. Prior to testing all rats were food deprived for 20 hours. For the test, each rat was presented with a ceramic bowl that contained either 15g of a familiar food (rat chow) or 15g of a novel food (TestDiet (TD) pellets, 5TUL 45mg); 3.4 kcal/g; 21% protein, 13% fat, 67% carbohydrate).

All rats were habituated to transport to the behavioral chamber room, and to the ceramic bowls, at least 24 hours prior to testing. The weight of all foods was measured following the end of testing to determine how much was consumed. Body weights for all rats were taken in the morning of test day. Average body weights were calculated for each group. All consumption data are presented as the number of grams consumed per 100 grams body weight.

### Histological Procedures

Rats were perfused 90 minutes after start of testing and brains were harvested. Rats were briefly anesthetized with isoflurane (5%; Baxter Healthcare Corporation, Deerfield, IL) and then given a lethal dose of Fatal-Plus (0.1mL/100g body weight, administered intraperitoneally; Vortech Pharmaceuticals; Dearborn, MI). Rats were then transcardially perfused with 0.9% saline followed by 4% paraformaldehyde in 0.1 M borate buffer. Brains were extracted and post-fixed overnight in a solution of 12% sucrose dissolved in the perfusion liquid, then rapidly frozen in hexanes cooled in dry ice and stored at − 80°C. Brains were sliced in 30-μm sections using a sliding microtome and collected into four adjacent series.

The first series was stained using standard immunohistochemical procedures for concurrent visualization of Fos and FluoroGold. Free-floating tissue sections were incubated in a blocking solution for 1 h at room temperature to minimize nonspecific binding. The blocking solution contained 0.02M potassium phosphate-buffered saline (KPBS), 0.3% Triton X-100 (Sigma-Aldrich), 2% normal goat serum (S-1000; Vector Laboratories, Burlingame, CA), and 10% non-fat milk (M-0841; LabScientific, Livingston, New Jersey). Then, the tissue was incubated with the primary antibody, anti-FluoroGold raised in rabbit (1:20,000, AB153-I, EMD Millipore, Billercia, MA) in the blocking solution for 72 h at 4°C. The tissue was rinsed in KPBS then incubated with the secondary antibody, biotinylated goat anti-rabbit IgG (1:500; BA-1000; Vector Laboratories) in the blocking solution for 45 min. Subsequently, the tissue was rinsed in KPBS then reacted with avidin–biotin complex (ABC solution; PK-6100; Vector Laboratories) for 45 min. To improve specific binding, this was followed by rinses in KPBS, a second 30 min incubation in the secondary antibody solution, rinses in KPBS, a second 30 min incubation in the ABC solution, and additional rinses in KPBS. To produce a color reaction (orange-brown), the tissue was incubated in a DAB (3.3’-diaminobenzidine) solution (SK-4100; Vector Laboratories) for 1–2 min with constant, manual agitation. The tissue then underwent a second round of staining to label Fos, using the same procedure above, but with primary antibody anti-c-Fos raised in guinea pig (1:60,000, 226 308, Synaptic Systems, Gottingen, Germany) and secondary antibody biotinylated goat anti-guinea pig IgG (1:500; BA-7000–1.5; Vector Laboratories). To produce a color reaction (gray-black), the tissue was incubated in a Nickel-intensified DAB solution (SK-4100; Vector Laboratories) for 1–2 min with constant, manual agitation. Stained tissue was then mounted onto SuperFrost Plus slides (Fisher Scientific, Pittsburgh, PA) and air- dried, followed by drying in an oven at 45°C overnight. Tissue was then dehydrated through graded alcohols, cleared in xylenes, and coverslipped with DPX (13512; Electron Microscopy Sciences, Hatfield, PA).

The second series was collected into KPBS solution, mounted onto gelatin-subbed slides, and stained with thionin for identification of cytoarchitectonic borders of brain structures, as defined in Swanson’s rat brain atlas (Swanson, 2018). The remaining series were collected into trays containing a cryoprotectant solution (0.025 M sodium phosphate buffer with 30% ethylene glycol and 20% glycerol) and stored at – ss20°C for later use. Brain perfusions, collection, slicing, and length of storage were counterbalanced across training conditions.

### Image Acquisition & Analysis

#### Statistical Analysis

Following arrival, males gained weight faster than females, resulting in body weight differences during testing. Therefore, all consumption results are reported as grams consumed per 100 grams of body weight ([food consumed(g)/body weight(g)]X100).

Consumption results were analyzed using a between-subjects 2-way univariate ANOVA for sex and testing condition. Differences in activation of CEA-projecting neurons (indicated by percentage of double-labelled neurons) for all regions of interest were analyzed using a between-subjects 2-way multivariate ANOVA for sex and testing condition. Differences in number of inputs from each region of interest to the CEA (calculated as proportion of sum of total inputs from ILA, PVTp, AId, BST, and CA1) was analyzed using a repeated measures ANOVA for region. All significant interactions were followed by Bonferroni *post hoc* analyses. A value of p < 0.05 was considered significant for all analyses, except for *post-hoc* analyses in which Bonferroni adjusted alpha level was used (p = 0.05/3 = 0.017). Data were analyzed for normality using Shapiro-Wilk test.

After exclusions for incorrect FG placement or insufficient deposit size, and tissue damage, final n’s for familiar and novel conditions for each brain region were as follows: ILA familiar condition n = 13, novel condition n = 11; AId familiar condition n = 12, novel condition n = 11; PVTp familiar condition n = 12, novel condition n = 10; BSTov/ju, familiar condition n = 14, novel condition n = 14; CA1 familiar condition n = 15, novel condition n = 14.

## Results

### Consumption

#### Retrograde Tracer Injections

**Figure 3:**
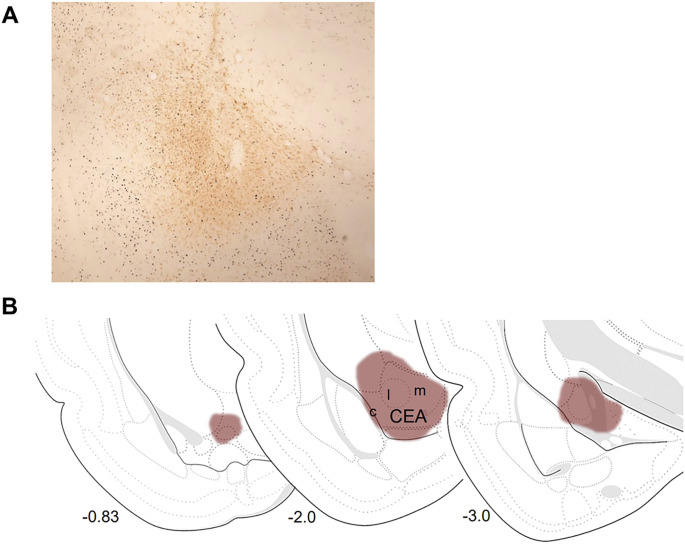
Retrograde tracer injection in the central nucleus of the amygdala (CEA) **A)** Image is a representative injection in the central nucleus of the amygdala taken at 4x magnification **B)** Illustration of the rostrocaudal extent of injection spread in same representative animal. Injection spread placed onto rat brain templates from Swanson rat brain atlas (2018) indicating locations of the medial (m), lateral (l), and capsular (c) subregions of the central nucleus.

In all animals included in analysis injections were centered in caudal CEA, with majority of injections centered within the lateral subregion and ventral portion of the medial subregion. The spread of injections encompassed the remainder of medial CEA and capsular CEA. The rostrocaudal extent of injections were comprehensive for majority of animals, with most spreading from the atlas level 22 (+ 0.83) to level 30 (−3.5). See Fig. 3 for representative injection image and illustration of injection spread.

### Retrograde Tracer Distribution

#### Proportion of Pathways to the Central Amygdala

##### Fos Induction in the CEA-projecting Neurons

###### Posterior Paraventricular Thalamus

**Figure 4:**
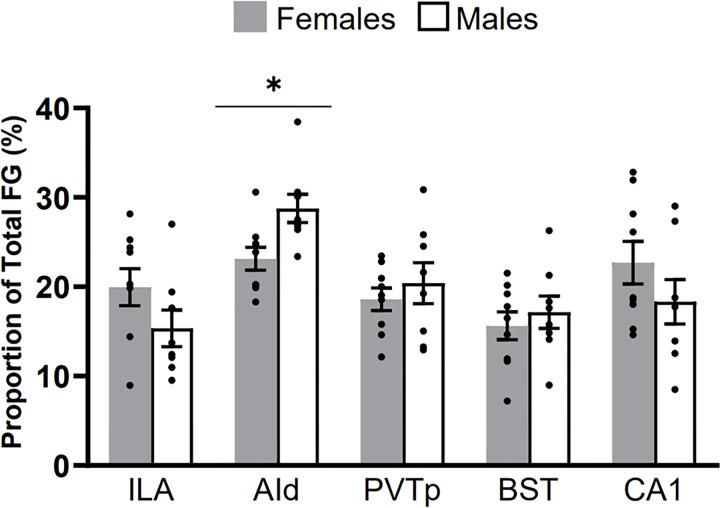
Proportion of inputs to the CEA, from the ILA, AId, PVTp, BST, and CA1 for males and females. The y-axis shows the percent of the total number of FluoroGold (FG) labeled neurons summed across the five regions. Asterisk indicates p<0.05.

The PVTp of rats tested in the novel condition had a greater percentage of FG neurons that were double labelled for Fos (FG + Fos) compared to the familiar condition (Fig. 4). A two-way ANOVA with the factors of sex and testing condition found a main effect of condition (F(1,18) = 6.523, p = 0.02), but no main effect of sex (F(1,18) = 1.487, p = 0.238) or a significant interaction of factors (F(1, 18) = 0.054, p = 0.819).

### Infralimbic Cortex

**Figure 5:**
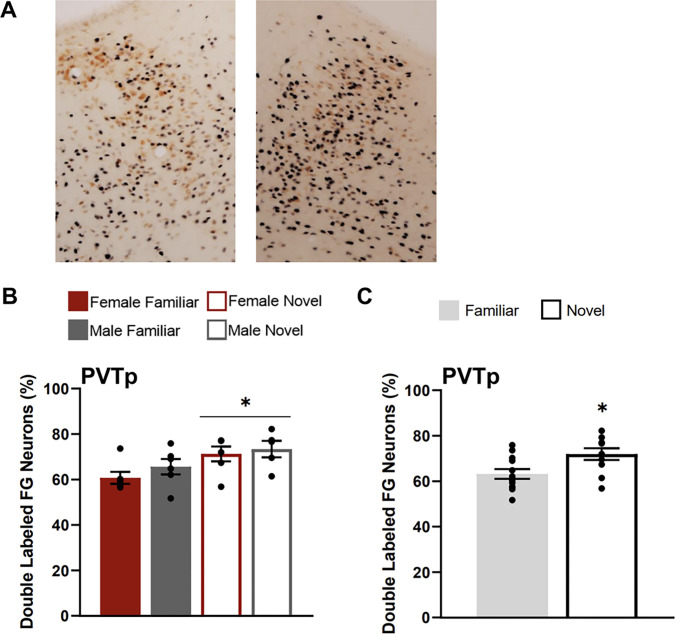
Fos induction in the PVTp neurons that project to the CEA**. A)** Images show tissue stained for Fos (black) and FluoroGold (FG; orange) in the PVTp (atlas level 31) for familiar (left) and a novel condition (right). Both animals were females. **B)** Percentage of PVTp FluoroGold neurons double labelled for Fos for each testing condition. **C)** Percentage of PVTp FluoroGold neurons doubled labelled for Fos collapsed across sexes for each condition (familiar & novel). Asterisk indicates p<0.05.

In the ILA, there were no differences between testing groups in the percentage of FG neurons that were double labelled for Fos (Fig. 5). A two-way ANOVA with the factors of sex and testing condition yielded no main effects of condition (F(1,20) = 1.202, p = 0.286), sex (F(1,20) = 0.839, p = 0.371) or interaction of these factors (F(1,20) = 0.278, p = 0.604).

**Figure 6:**
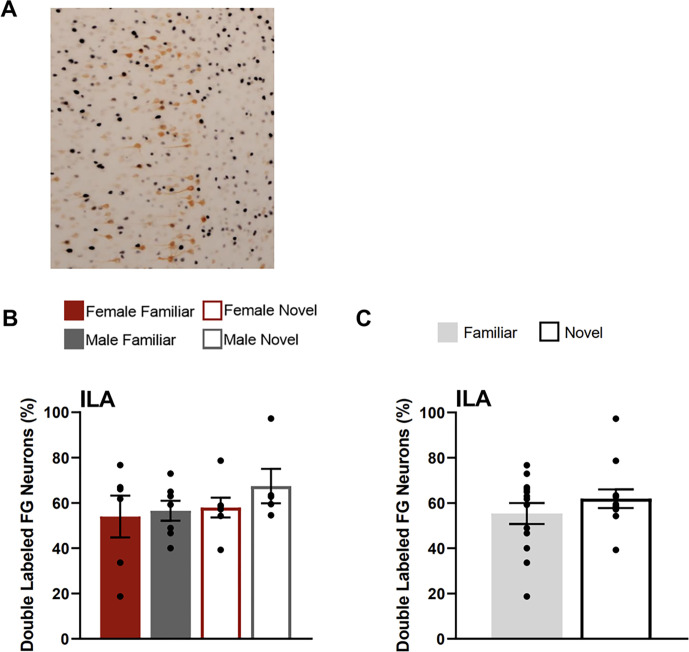
Fos induction in the ILA neurons that project to CEA. **A)** Image shows tissue stained for Fos (black) and FluoroGold (FG; orange) in the ILA (atlas level 9) for familiar condition animal (female). **B)** Percentage of FluoroGold neurons with the ILA double labelled for Fos for each testing condition. **C)** Percentage of ILA FluoroGold neurons doubled labelled for Fos collapsed across sexes for each condition (familiar & novel).

There were no differences in the percentage of FG neurons that were double labelled for Fos in the AId between testing groups (Fig. 6). A two-way ANOVA with the factors of sex and testing condition yielded no main effects of condition (F(1,19) = 0.824, p = 0.375), sex (F(1,19) = 0.608, p = 0.445), or interaction of these factors (F(1,19) = 0.001, p = 0.977).

### Bed Nucleus of the Stria Terminalis

**Figure 7:**
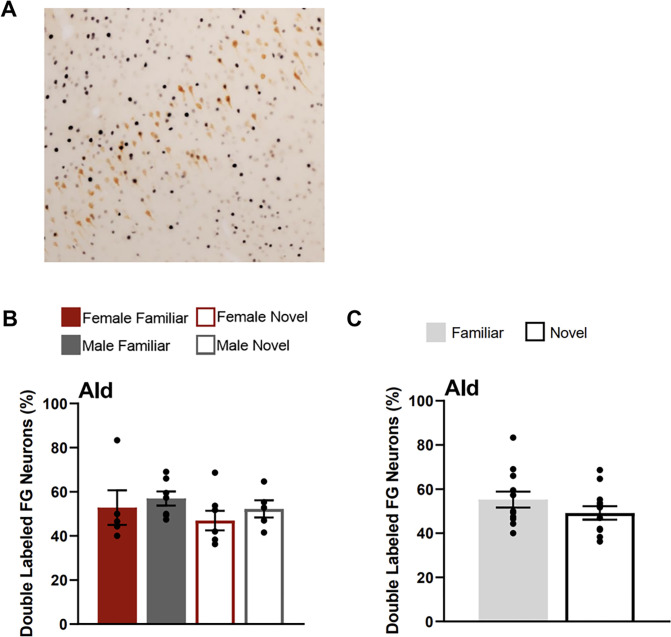
Fos induction in the AId neurons that project to CEA. **A)** Image shows tissue stained for Fos (black) and FluoroGold (FG; orange) in the AId (atlas level 14) for a novel condition animal (female).**B)** Percentage of AId FluoroGold neurons double labelled for Fos for each testing condition. **C)** Percentage of AId FluoroGold neurons doubled labelled for Fos collapsed across sexes for each condition (familiar & novel).

There were no differences in the percentage of FG neurons that were double labelled for Fos in the BSTov/ju between testing groups (Fig. 7). A two-way ANOVA with the factors of sex and testing condition yielded no main effects of condition (F(1,24) = 0.126, p = 0.725) sex (F(1,24) = 0.235, p = 0.632), or interaction of these factors (F(1,24) = 1.861, p = 0.185).

### Hippocampal ventral field CA1

**Figure 8:**
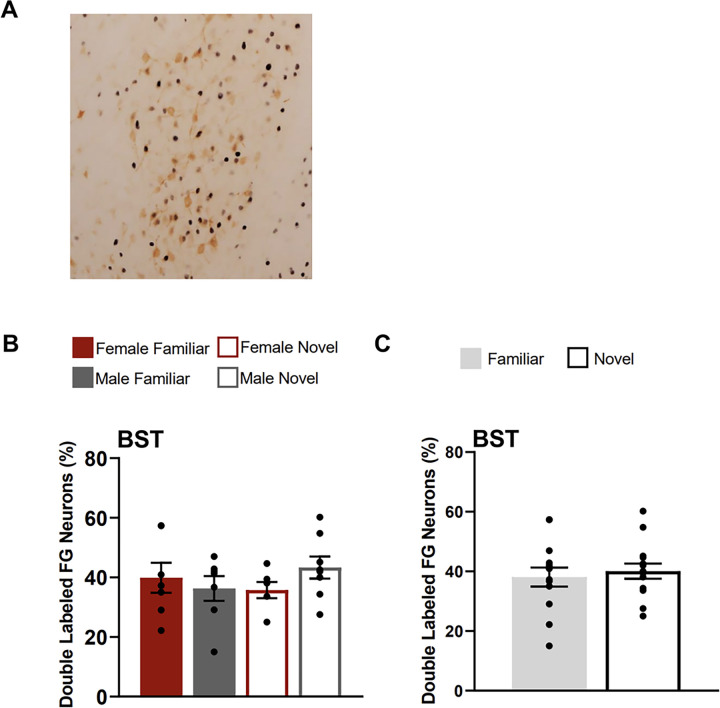
Fos induction in the BST neurons that project to CEA. **A)** Image shows tissue stained for Fos (black) and FluoroGold (FG; orange) in the BST (atlas level 19) for a familiar condition animal (female). **B)** Percentage of BST FluoroGold neurons double labelled for Fos for each testing condition**. C)** Percentage of BST FluoroGold neurons doubled labelled for Fos collapsed across sexes for each condition (familiar & novel).

The CA1 of rats tested in the novel condition had a greater percentage of FG neurons that were double labelled for Fos (FG + Fos) compared to the familiar condition (Fig. 8). A two-way ANOVA with the factors of sex and testing condition revealed a main effect of condition (F(1,25) = 8.585, p = 0.007), but no main effect of sex (F(1,25) = 2.631, p = 0.117) or a significant interaction of factors (F(1,25) = 0.105, p = 0.748).

## Discussion

The present study examined the recruitment of specific CEA pathways during food consumption under novel conditions. Our previous findings identified the CEA as a central hub for mediating consumption during novelty processing (Greiner et al., 2023). Here, we analyzed the recruitment of CEA-projecting neurons within the ILA, PVTp, AId, BSTov/ju, and ventral CA1. Injection of the retrograde tracer FluoroGold (FG) into the CEA prior to behavioral testing

### Food Consumption Under Novelty

During the food consumption tests, animals in the novel condition ate less than animals in the familiar condition and males and females had similar consumption patterns under the same conditions. sConsumption results in the current study aligned with the behavioral findings reported in Greiner & Petrovich (2020), such that novel foods and contexts induced lower food intake. Similar behavior of males and females during the first presentation of a novel food was also consistent with previous results, as sex differences in food consumption did not occur until later habituation. A distinction from the previous behavioral paradigm was that the current investigation compared the effect of novel context and novel food simultaneously, rather than either stimulus individually.

### Distribution of Inputs to the Central Nucleus of the Amygdala

Here we identified the FG distribution within five regions that send major inputs to the CEA and quantified the contribution from each region. We compared the relative contribution of each CEA-projecting region to the total inputs from all five regions analyzed and found that the greatest proportion of inputs to the CEA came from the AId (approximately 25%). The second highest proportion of total inputs originated in the CA1 (approximately 20%). The ILA, PVTp, and BSTov/ju contributed similar portions of inputs to the CEA (approximately 18% each). Males and females had similar proportions of inputs to the CEA from these areas.

### Comparisons with Prior Tract Tracing Studies

The current findings are in general agreement with prior anterograde and retrograde tract tracing results. Our analysis of FG labelling within the PVTp, ILA, AId, BSTov/ju, and ventral CA1 confirmed projections from these regions to the CEA and found novel data in regard to inputs from AId.

The PVTp is known to send strong projections to the CEA, which is its most densely innervated target within the amygdala (Li and Kirouac, 2008; Vertes and Hoover, 2008; Unzai et al., 2015). Previous tracing studies identified that anterograde tracer injection into the PVTp resulted in dense labelling in posterior CEA and weaker labeling in anterior CEA (Li and Kirouac, 2008). The FG injection sites in the current study were centered in the posterior CEA and encompassed the lateral and capsular subregions where most PVTp projections terminate (Li and Kirouac, 2008).

The ILA is the major medial prefrontal cortex input to the CEA (McDonald et al., 1996; McDonald 1998). Majority of ILA inputs to the CEA terminate within its capsular and medial subregions (Hurley et al., 1991; Bienkowski & Rinaman, 2012; McDonald 1998), which were targeted in the current study. In agreement with these findings, we observed FG labeled neurons within layer 3 and 5 of the ILA (Hurley et al., 1991). We additionally observed sparse labelling within layer 6, which was not observed in previous studies, and may be due to the larger injection size in our experiment.

Within the BST, we observed FG labelling in both the BSTju and BSTov, in agreement with prior work. Both nuclei send dense projections to the medial portion of the CEA with lighter projections to the capsular CEA (Dong et al., 2000; Dong et al., 2001). The key difference between projections from the two nuclei is that BSTov sends moderate projections to the lateral portion of the CEA (Dong et al., 2001), where the BSTju does not (Dong et al., 2000). Our FG injection contained the lateral, medial and capsular CEA, which prevented us from distinguishing between the projections to separate CEA subregions.

Within the CA1, we found that projections to the CEA originated in the ventral portion of CA1 and did not observe any substantial FG labelling in dorsal CA1. This is in agreement with previous work that examined anterograde tracers across dorso-ventral CA1. Anterograde injections into the ventral portion of the CA1 resulted in labelling within all parts of the CEA and was most robust in the capsular subregion, while these projections were not observed in injections to the more dorsal portions of CA1 (Cenquizca & Swanson, 2007).

Projections from AI to CEA are also well established. Several anatomical tract tracing studies using both retrograde and anterograde labelling have found connections between AI regions and the CEA (McDonald 1998; Bienkowski & Rinaman, 2012). Our study provides additional anatomical specificity to these connections. In the current study, the FG labeled neurons were distributed selectively within the AId. Prior work investigated anterior and posterior AI regions, which contains AId (Allen et al., 1991; Shi & Cassell, 1998). The FG neurons within the AId were concentrated in layer 2–3, with sparse labelling in layer 1 and Projections to CEA from AId layer 2–3 implicates early sensory information processing.

### Recruitment of the CEA Inputs

Analyses of Fos induction in CEA-projecting neurons (FG + Fos) within the ILA, PVTp, and AId, BSTov/ju, and CA1, revealed that the projections from two areas were selectively activated in animals in the novel condition compared to familiar controls. There was greater activation of the PVTp neurons that project to the CEA and of the CA1 neurons that project to the CEA. Neurons within the AId, ILA, and BSTov/ju that send projections to the CEA were similarly activated in all groups.

### Inputs from the Posterior Paraventricular Nucleus of the Thalamus

We found greater recruitment of the CEA-projecting neurons within the PVTp for rats that consumed food in the novel condition compared to the familiar condition in both sexes. Projections from the PVT to CEA are largely associated with driving fear and anxiety behaviors (Chen & Bi, 2019; Do Monte et al., 2015; Penzo et al., 2015). The novelty-induced states in tasks like ours are considered to model anxiety (Ramaker & Dulawa, 2017), implicating anxiety circuits as mediators of food avoidance under novelty. Greater recruitment of the PVTp to CEA pathway may drive greater avoidance behavior, which would enhance cessation of feeding in the novel condition. Previous work has found that stimulation of neurons in the PVTp that project to the CEA reduced time in open arms on an elevated plus maze, which is typically interpreted as greater anxiety responding (Chen & Bi, 2019; Pliota et al., 2020). Similarly, stimulation of PVTp increased avoidance of the center of an open field (Li et al., 2009; Li et al., 2010).

In contrast to the current findings of selective recruitment of the CEA-projecting PVTp neurons under novelty, the patterns of overall Fos induction in the PVTp were less consistent. Our prior study found greater Fos induction in the PVTp in the novel context condition, however the effect was slightly above the significance (p = 0.051) (Greiner & Petrovich, 2023), while another study did not find changes in overall PVTp activation during feeding suppression in a novel context in mice (Cheng et al., 2018). Collectively, these could suggest that the CEA- and non-CEA projecting neurons within PVTp are distinctly recruited in the novel and control conditions.

### Inputs from the Ventral Hippocampal Field CA1

We found greater recruitment of the CEA-projecting neurons within the ventral CA1 for rats that consumed food in the novel condition compared to the familiar condition. The recruitment of the CA1-CEA pathway was similar for both males and females within each condition. Interestingly, majority of the CA1 inputs to the CEA have been shown to synapse on somatostatin-expressing (CEA^SST^) neurons in mice (Fu et al., 2020). The CEA^SST^ neurons are crucial in fear conditioning and memory recall (Li 2019). Nevertheless, whether the CA1 neurons recruited in the current study, in rats, synapse selectively on the CEA^SST^ neurons and how that pathway may mediate defensive responding to a potential threat of uncertainty in the novel condition remains to be established.

### Inputs from the Dorsal Agranular Insular Cortex

The current study found similar recruitment of the CEA-projecting neurons within the AI across groups. This finding was unexpected, as we predicted that the taste differences between the novel and familiar foods would differently activate inputs from the AId to the CEA. The insular cortex, including the AId, processes taste and visceral information (Jasmin et al., 2004; Chen et al., 2011; for review see: Moraga-Amaro & Stehberg, 2012; Boughter & Fletcher, 2021) and controls feeding behavior (Giacomini et al., 2022). The AId was activated during exposure to a novel taste (Koh et al., 2003; Bermudez-Rattoni, 2014). Insular cortex connections to the CEA mediate appetitive and aversive responding (Haaranen et al., 2020; Gehrlach et al., 2019; Schiff et al., 2018; Wang et al., 2018; Zhang-Molina et al., 2020). Importantly, activation of the AId-CEA pathway suppressed consumption and appetitive behavior, even in food deprived mice (Zhang-Molina et al., 2020). In the current study, the AId-CEA was recruited similarly, even though rats consumed different amounts of two distinct tasting foods in the novel and familiar conditions. It is possible that distinct CEA-projecting neurons within the AId were recruited in each condition, however, the method used in the current study could not determine if the same or different cell types contributed to the AId-CEA pathways across conditions. Furthermore, the same AId neurons could innervate multiple cell types within the CEA (Zhang-Molina et al., 2020). The CEA contains neurons that both drive (Douglass et al., 2017) and suppress feeding (Cai et al., 2014) and the AId excitatory inputs to the CEA do not target a single cell-type (Schiff et al., 2018; Cai et al., 2014; Douglass et al., 2017; Zhang-Molina et al., 2020).

### Inputs from the Infralimbic Cortex

The current study found similar recruitment of the CEA-projecting neurons within the ILA across groups. The ILA is well known for extinction learning and memory recall, particularly in regard to fear conditioning (Milad & Quirk, 2002; Burgos-Robles et al., 2009; Rozeske et al., 2015), and stimulation of projections from the ILA to CEA were shown to inhibit anxiety-like responding (Chen et al., 2021). The ILA also controls feeding behavior as a part of the ventromedial prefrontal cortex (Petrovich et al., 2007; Anderson & Petrovich 2018; Giacomini et al., 2022; Cole et al., 2020). Our previous study (Greiner et al., 2023) found an increase in overall ILA activation when rats consumed food in a novel context. The present findings indicate that the increased ILA activity we observed previously may not have been due to a greater recruitment of the CEA projecting neurons, but possibly due to recruitment of local inhibitory neurons or the ILA neurons that project other targets. In that regard, ILA projections to the nucleus accumbens have been shown to regulate avoidance behavior (Schwartz et al., 2017). However, it is unknown if the CEA-projecting neurons within the ILA are a uniform population and if they target different CEA neurons. Given the ILA role in the control of aversive and appetitive behaviors, different and potentially competing ILA-CEA pathways could be recruited during habituation to novelty, which the quantitative analysis in the current study could not capture.

### Inputs from the Bed Nucleus of the Stria Terminalis

In the current study, there was similar recruitment of the CEA-projecting neurons with the BSTov/ju across groups. This finding was unexpected given the well-known role in stress, including responding to unpredictable stimuli (Walker et al., 2009), of the BSTov, and its neurons that express corticotropin releasing hormone/factor (CRH/CRF). The BSTov and the lateral CEA share anatomical and functional similarities, particularly in regard to the co-expression of CRH/CRF in GABAergic neurons in both regions (Ju & Swanson, 1989; Ju et al., 1989; Day et al., 1999; Marchant et al., 2007, McCullough et al., 2018). However, the current study did not examine specific connections of the CRH/CRF neurons, which may be the reason for the observed similar Fos induction in the BSTov/fu-CEA pathways. Different BSTov neurons (protein kinase C-delta) have been shown to suppress feeding during physiological stress of inflammation, primarily by projections to other BST nuclei, in order to influence downstream projections to the lateral hypothalamus (Wang et al., 2019). Therefore, complex BST microcircuits may have been recruited, during feeding under novelty, including potentially competing CRH/CRF and other BSTov/fu-CEA pathways that would have not been captured in the current study.

In summary, the current findings provide evidence that CEA inputs are recruited differently during food consumption under novelty. We identified specific recruitment of the neurons within the PVTp and ventral hippocampal field CA1 that send direct inputs to the CEA that corresponded to the behavioral differences in consumption under novel and familiar conditions. These results suggests that the PVTp-CEA and ventral CA1-CEA pathways are important for feeding inhibition during novelty processing. These findings add to our understanding of the neural circuit mechanisms underlying the control of food consumption under uncertainty and point to potential sites where malfunction could cause excessive food avoidance and psychopathology.

## Figures and Tables

**Figure 1:**
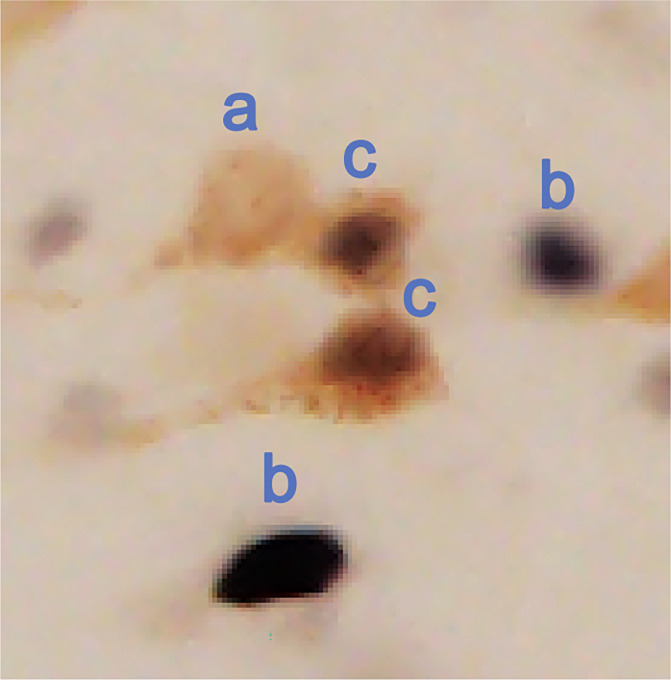
Image shows representative types of labeled neurons. Lowercase letters indicate a representative of each type of labeled neuron: single-labeled FluoroGold (a), single-labeled Fos (b), and double-labeled FG and Fos (c).

**Figure 2:**
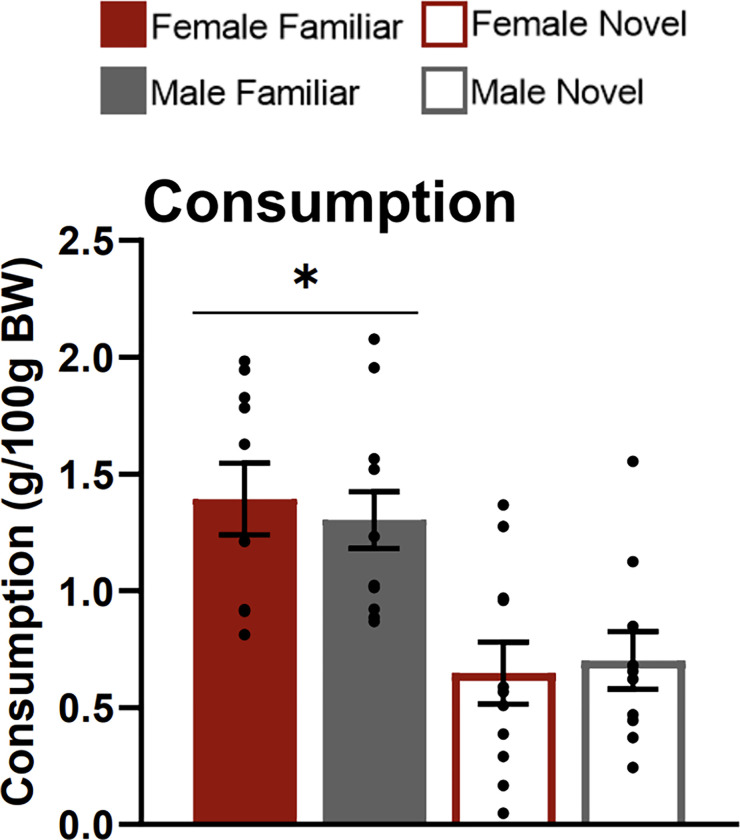
Food consumption test. The graph shows the amounts of each food that subjects in each testing condition consumed, expressed as grams per 100 grams of their body weight (BW). Asterisks indicate p<0.05.

**Figure 9:**
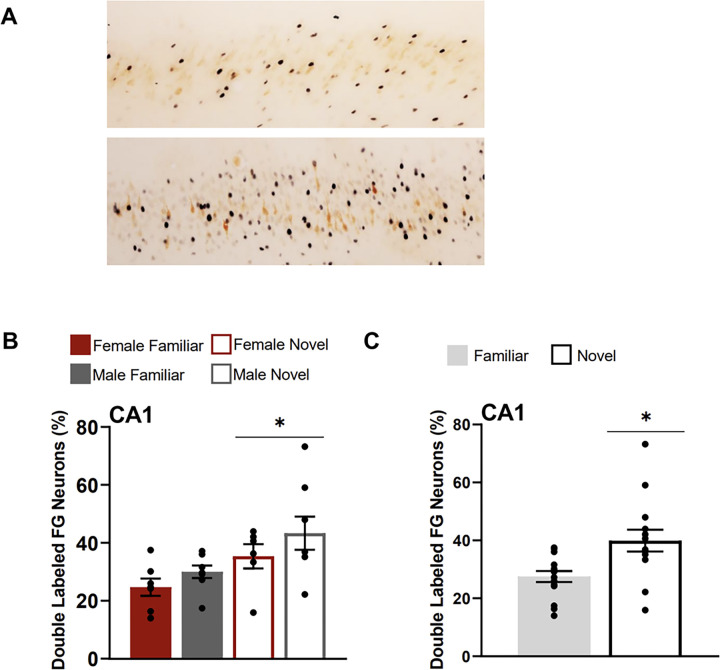
Fos induction in the ventral CA1 neurons that project to the CEA. **A)** Images show tissue stained for Fos black) and FluoroGold (FG; orange) in the CA1 (atlas level 32) for familiar (top) and a novel condition (bottom). Both animals were females. **B)** Percentage of CA1 FluoroGold neurons double labelled for Fos for each testing condition**. C)** Percentage of CA1 FluoroGold neurons doubled labelled for Fos collapsed across sexes for each condition (familiar & novel). Asterisk indicates p<0.05.

## Data Availability

The data that support the findings of this study are available from the corresponding author upon reasonable request.
